# Supplementary material for: Secretory granule protein chromogranin B (CHGB) forms an anion channel in membranes
Source: Life Sci Alliance. 2018 Sep 24;1(5):e201800139. doi: 10.26508/lsa.201800139 (PMC6238609; doi:10.26508/lsa.201800139)
Supplement: Supplementary file 1 [file LSA-2018-00139_TableS1.doc]

**Table S1 Proteins identified by Mass Spec and proteomic analysis of the CHGB band**

| **Protein name** | **Acc. number** | **Mol. Weight (Da)** | **Peptide counts** | **Peptide seq.** | **score** |
| --- | --- | --- | --- | --- | --- |
| **Likely candidate proteins (based on MW)** | | | | | |
| Secretogranin-1(CHGB)  Light chain of kinesin  Junction plakoglobin(jup)  Vacuolar protein sorting-associated protein 35(Vps35)  Embryonic poly(A)-binding protein (Pabpc1)  Cullin 3 (Protein providing scaffold for ubiquitin ligases)  ATPase, Ca++ transporting, (Atp2a3) Minichromosomal  Maintenance Complex Component 6 (Mcm6)  TNF Receptor Associated Protein 1(Trap1)  Kinesin member protein (Kif5c)  Heat shock cognate protein (Hspa8)  Cullin-associated NEDD8-dissociated protein 1(Cand1)  Tetratricopeptide repeat protein 21B (Ttc21b)  Suppressor of Ty 16 (Supt16h)  Serine/threonine-protein phosphatase 2A (Ppp2r1a) | P16014  D3YXZ3  Q02257  Q9EQH3  A2A5N3  Q6ZQ84  B1ATS4  Q3ULG5  F6YP65  Q8CHF1  Q504P4  Q6ZQ38  Q0HA38  G3X956  Q76MZ3 | 78036  68534  82490  92453  67733  91846  08900  90697  80323  113251  68964  137955  152294  120334  66079 | 61  2  4  2  1  3  1  1  1  1  1  5  1  1  3 | APQLDLK  TLHNLVIQYASQGR  ALMGSPQLVAAVVR  AELAELPLR  VGTKPLYVALAQR  FLPSPVVIKK  SAAEMVLSDDNFASIVAAVEEGR  IQETQAELPR  GVVDSEDIPLNLSR  LYLVDLAGSEK  IINEPTAAAIAYGLDK  LDDDSER  LIDLLR  FPGEFMK  LSTIALALGVER | 15382  155  122  49  55  67  73  82  83  84  97  122  43  40  227 |
| **Unlikely candidate proteins (based on MW)** | | | | | |
| Psmc2 (Proteasome 26S Subunit, ATPase 2)  ADP/ATP translocase 1 (Slc25a4)  Ribosomal protein S5 (Rps5)  GTP-binding nuclear protein (Ran)  Ribosomal protein (Rps3)  Member RAS oncogene family(Rab1)  Serine protease (Prss1)  Cardiotrophin-Like Cytokine Factor 1(Clcf1) | Q8BVQ9  P48962  Q91V55  P62827  D3YV43  Q5SW88  Q9Z1R9  Q3TM06 | 53289  33111  23033  24579  21209  22586  26802  24385 | 5  3  5  4  3  3  1  1 | SVCTEAGMFAIR  YFPTQALNFAFK  QAVDVSPLR  FNVWDTAGQEK  ELAEDGYSGVEVR  LQIWDTAGQER  LGEHNINVLEGNEQFIDAAK  LGAETLPR | 384  250  162  115  99  90  67  43 |
