# Supplementary material for: Secretory granule protein chromogranin B (CHGB) forms an anion channel in membranes
Source: Life Sci Alliance. 2018 Sep 24;1(5):e201800139. doi: 10.26508/lsa.201800139 (PMC6238609; doi:10.26508/lsa.201800139)
Supplement: Supplementary file 2 [file LSA-2018-00139_TableS2.doc]

**Table S2 Proteins identified in two contaminating** bands

| **Band #** | **Protein name** | **Acc. number** | **Mol. Weight (Da)** | **Peptide count** | **Peptide seq.** |
| --- | --- | --- | --- | --- | --- |
| Contaminant 1 | Chromogranin B  Ubiquitin carboxyl-terminal hydrolase 48  AT-rich interactive domain-containing protein 5B  Phosducin-like protein  Kallikrein related-peptidase 12  ER membrane protein complex 8  VWFA and cache domain-containing protein 1  HMG domain containing Protein3 (Hmgxb3)  Olfactory receptor  Growth hormone receptor  Potassium voltage-gated channel (KQT member 5)  Chromatin remodeling Protein (Cecr2)  IgG Fc binding Protein (Fcgbp)  Trypsinogen (Prss3 protein) (Fragment) | Q3TY66  F6VSP0  ARI5B  A2AVP3  B2RVZ0  Q8C988  CAHD1  G3X9M3  Q8VGG1  GHR  E9Q9F1  E9Q2Z1  E9Q0B5  B9EJ35 | 77969  17,484.20  131,840.70  34,407.60  25,886.80  23,290.30  143,812.60  140,729.50  34,360.00  72,785.40  102,261.00  161,530.90  275,211.10  26,134.50 | 9  1  1  1  1  1  1  1  1  1  1  1  1  1 | EDAGAPVEDSQGQTK  EENEALHK  QENNTQENENK  FKQLETEQR  GIPGVYTK  GASPNQVAEK  LSTTVNSR  VCPHQVVCGSK  EMINAIKK  SAGILGAK  SASANISR  AASSGDDQSR  ISVINGGSK  TLNNDIMLIK |
| Contaminant 2 | Chromogranin B  Zinc finger protein 809  Kallikrein related-peptidase 12  Small EDRK-rich factor 2  VWFA and cache domain-containing protein 1  Potassium voltage-gated channel (KQT member 5)  IgG Fc binding Protein (Fcgbp) | Q3TY66  Q8BIL4  Q9CV76  D6RIP7  CAHD1  E9Q9F  E9Q0B | 77,969.00  19,742.60  25,886.80  6,838.20  143,812.60  102,261.00  275,211.10 | 5  1  1  1  1  1  1 | APQLDLK  NHMNVQNAR  GIPGVYTK  QSFSRQAQR  LSTTVNSR  SASANISR  ISVINGGSK |

**NOTE: Multiple CHGB-related peptides that are identified and shared between both bands.**
