# Supplementary material for: Secretory granule protein chromogranin B (CHGB) forms an anion channel in membranes
Source: Life Sci Alliance. 2018 Sep 24;1(5):e201800139. doi: 10.26508/lsa.201800139 (PMC6238609; doi:10.26508/lsa.201800139)
Supplement: Supplementary file 3 [file LSA-2018-00139_TableS3.doc]

**Table S3**: Parameters estimated for the reconstituted CHGB vesicles

| Average diameter:  Capacitance coefficient:  Surface area of each lipid (PC) molecule (White and King, 1985):  Average number of lipid molecules per 100 nm vesicle:  Average vesicle concentration (0.4 mg/ml lipid):  Volume inside a 100 nm vesicle:  Number of K^+^ or Cl^-^ ions per 100 nm vesicle:  Number of Cl^-^ ions (*W*) moved to reach a stable Nernst potential:  Average [Cl^-^] outside when a stable Nernst potential was reached:  Steady-state Nernst potential before valinomycin addition: | 100 nm  ~1 µF/cm^2^  ~70 Å^2^  ~8 x 10^4^  ~5.0 nM  ~0.41 aL  7.4 x10^4^  ~580  ~2.6 µM  ~ +295 mV |
| --- | --- |
